# Evaluating plasma carboxylesterase 1 as a protein biomarker for pharmacokinetics and metabolic dysfunction–associated steatotic liver disease

**DOI:** 10.1016/j.dmd.2026.100307

**Published:** 2026-04-29

**Authors:** Sun Min Jung, Hui Yu, Xinwen Wang, Lucy Her, Hao-Jie Zhu

**Affiliations:** 1 Department of Pharmaceutical Sciences, University of Michigan, Ann Arbor, Michigan; 2 Department of Pharmaceutical Sciences, Northeast Ohio Medical University, Rootstown, Ohio; 3 Department of Global PK/PD and Pharmacometrics, Eli Lilly and Company, Indianapolis, Indiana; 4 Department of Clinical Pharmacy, University of Michigan, Ann Arbor, Michigan

**Keywords:** Carboxylesterase 1, Biomarker, Pharmacokinetics, Metabolic dysfunction–associated steatotic liver disease, Machine learning

## Abstract

Carboxylesterase 1 (CES1) is the most abundant hepatic hydrolase responsible for metabolizing many clinically important medications. Hepatic CES1 also plays a role in regulating lipid homeostasis. This study aimed to determine whether plasma CES1 protein levels reflect hepatic CES1 abundance and serve as a biomarker of the pharmacokinetics of CES1-substrate drugs and of metabolic dysfunction–associated steatotic liver disease (MASLD). We quantified CES1 protein levels in matched human livers and plasma from adults with biopsy-confirmed MASLD (*n* = 17), in normal liver tissues (*n* = 6), and in plasma from healthy volunteers enrolled in an enalapril pharmacokinetic study (*n* = 19). First, we found no correlation between hepatic and plasma CES1 levels in matched liver and plasma samples from patients with MASLD. Second, plasma CES1 correlated with enalaprilat exposure, although the correlation did not reach statistical significance. Third, plasma CES1 concentrations were approximately 8-fold higher in patients with MASLD than in healthy subjects (median 141.9 vs 16.2 ng/mL), whereas hepatic CES1 levels were comparable between MASLD and normal liver tissues. Moreover, plasma CES1 concentrations rose stepwise across histologic NAFLD activity score (NAS) strata (healthy < NAS 1–4 < NAS ≥ 5). Fourth, the machine learning model, Lasso logistic regression, showed that the CES1-informed classifier consistently outperformed those based on clinical features in differentiating MASLD patients from healthy subjects and across disease severity (NAS 1–4 vs NAS ≥ 5). Together, these data position plasma CES1 as a potential biomarker for diagnosing MASLD and staging the disease.

**Significance Statement:**

The findings of this study indicate that plasma carboxylesterase 1 may act as a substrate-dependent biomarker for the pharmacokinetics of drugs metabolized by the enzyme. Additionally, this study highlights plasma carboxylesterase 1 as a potential noninvasive biomarker of metabolic dysfunction–associated steatotic liver disease/metabolic dysfunction–associated steatohepatitis that can complement existing diagnostic tools and provide a more comprehensive assessment of disease progression.

## 1 Introduction

Carboxylesterase 1 (CES1), the most abundant hepatic drug metabolizing enzyme, is responsible for the hydrolysis of numerous ester-containing therapeutics, endogenous lipids, and environmental substrates.^1^^,^^2^ Hepatic CES1 expression and activity vary markedly among individuals, contributing to interindividual variability in the pharmacokinetics (PK) and pharmacodynamics (PD) of CES1-substrate drugs. Numerous genetic and nongenetic regulators affect CES1 function.^2^ Among all *CES1* genetic polymorphisms examined to date, the G143E (rs71647871) variant, a *CES1* nonsynonymous loss-of-function variant, is the only polymorphism consistently demonstrated to affect the PK and PD of drugs metabolized by CES1.^3^ However, given its low minor allele frequency (<3%), the G143E genotype accounts for only a small fraction of PK variability, underscoring the need for additional noninvasive biomarkers of CES1 functional capacity.^4^

CES1 localizes primarily to the endoplasmic reticulum via a C-terminal HIEL endoplasmic reticulum retrieval sequence in the liver and can be released in the blood at low levels.^5^ A proof-of-concept study in healthy volunteers treated with the CES1 substrate drug methylphenidate showed that plasma CES1 concentrations inversely correlated with *d*-methylphenidate exposure, indicating plasma CES1 has the potential to serve as a biomarker of in vivo CES1 function.^6^ However, it remains unknown whether plasma CES1 predicts the PK of other CES1 substrates and correlates with hepatic CES1 abundance.

Direct evaluation of plasma-liver CES1 relationships requires matched plasma and hepatic tissue from the same individual. Because research biopsy in healthy subjects is not permissible, paired specimens are typically obtained from patients undergoing clinically indicated biopsy. Metabolic dysfunction–associated steatotic liver disease (MASLD), formerly known as nonalcoholic fatty liver disease (NAFLD) and its progressive form, metabolic dysfunction–associated steatohepatitis (MASH), represent common clinical scenarios where liver biopsy is indicated—typically when noninvasive assessments suggest steatohepatitis or advanced fibrosis, when clinical features are atypical, or when alternative diagnoses must be excluded.^7^, ^8^, ^9^ Assessing MASLD activity noninvasively remains challenging: widely used tests prioritize fibrosis (eg, fibrosis-4 index^10^ and enhanced liver fibrosis^11^) or apoptosis (eg, cytokeratin-18^12^), with limited ability to capture early metabolic stress or hepatocellular injury. Hepatic CES1 plays an important role in lipid homeostasis in the liver, and disease-related alterations in hepatocyte lipid handling could influence circulating CES1.^13^, ^14^, ^15^ Although previous studies have reported associations between circulating CES1 and fatty liver, these analyses lacked biopsy confirmation and did not evaluate correlations between plasma CES1 levels and the PK of CES1 substrates.^16^, ^17^, ^18^

Accordingly, this study evaluated plasma CES1 across 3 complementary contexts: (1) its relationship with hepatic CES1 abundance in paired liver-plasma samples from biopsy-confirmed MASLD; (2) its association with CES1-mediated drug metabolism using enalapril PK data from healthy adults; and (3) its utility as a noninvasive biomarker of histologic MASLD disease activity. Using absolute CES1 quantification, we related circulating CES1 to hepatic CES1 levels, enalapril PK, and MASLD, including the NAFLD activity score (NAS). We then benchmarked its diagnostic performance against routine laboratory measures by training the Lasso logistic regression classifier on 3 feature sets: plasma CES1 only, clinical labs only, and both combined.

## 2 Materials and methods

### 2.1 Paired liver and plasma samples from patients with MASLD

Paired liver biopsy and plasma samples from 17 patients with biopsy-confirmed MASLD were obtained through the Liver Tissue and Blood Repository in the Division of Gastroenterology and Hepatology at the University of Michigan. All specimens were stored in ALLprotect tissue reagent (QIAGEN) at –80 °C prior to analysis. Patients had a range of MASLD severity, as assessed by the NAS derived from liver biopsy reports. Deidentified clinical metadata provided by the repository included age at biopsy, sex, race/ethnicity, select laboratory values (eg, aspartate aminotransferase [AST], alanine aminotransferase [ALT], white blood cell [WBC] count), and relevant comorbidities extracted from medical records.

This study was approved by the University of Michigan Institutional Review Board (IRB #: HUM00201528) for secondary use of archived biospecimens and associated data, in accordance with institutional policies and the Declaration of Helsinki.

### 2.2 Normal human liver tissues

Six liver tissues were randomly selected from a collection of 153 normal liver samples obtained from the Cooperative Human Tissue Network. The donor pool included 50% males, 5 Caucasians, and 1 African American, with ages ranging from 1 to 83 years.

### 2.3 Plasma samples from healthy volunteers

Plasma samples from healthy adult volunteers were obtained from a previously published enalapril PK study.^4^ All participants were healthy, nonsmoking adults with no known hepatic or renal diseases at enrollment. Plasma samples were processed according to the original study protocol and stored at –80 °C until analysis. For the present study, baseline (predose) plasma samples were used for CES1 quantification. All samples were deidentified prior to analysis.

### 2.4 Preparation of human liver S9 fractions and protein quantification

Liver tissues were washed twice with ice-cold phosphate buffer (100 mM, pH 7.4) to remove ALLprotect tissue reagent and homogenized in 50 *μ*L of ice-cold phosphate buffer using a handheld pestle homogenizer in Eppendorf tubes. Homogenates were centrifuged at 9000*g* for 20 minutes at 4 °C, and the resulting supernatant was collected as the S9 fraction. S9 fraction samples were aliquoted and stored at –80 °C until analysis. Total protein concentrations of each human liver S9 fraction (HLS9) sample were quantified using the Qubit Protein Assay Kit (Thermo Fisher Scientific) according to the manufacturer’s instructions. S9 fractions were then diluted to a uniform concentration of 1 ng/μL total protein prior to CES1 quantification by ELISA.

### 2.5 Quantification of CES1 in plasma and HLS9 via ELISA

Plasma and HLS9 CES1 protein concentrations were measured using a commercial human CES1 ELISA kit (Boster Bio, catalog #EK1813). Measurements were performed in at least 3 independent experiments. The CES1 concentrations of the standard curves were adjusted to cover the wide interindividual variability in plasma CES1 concentrations. Human recombinant CES1 protein (BioLegend, catalog #794606, carrier-free) was used in place of the standards provided in the ELISA kit. The CES1 protein was diluted in 7% bovine serum albumin to prepare a stock CES1 solution at 1000 ng/mL. Serial dilutions were prepared in the Boster Bio sample diluent buffer to generate a 7-point calibration curve (1000, 500, 250, 125, 62.5, 31.25, and 15.625 ng/mL). The assay followed the protocol provided by the manufacturer, except that washes were performed in phosphate buffer containing 0.05% Tween-20. Absorbance was read at 450 nm, and CES1 concentrations were interpolated from the standard curve using 4-parameter logistic regression, with baseline correction by subtracting absorbance in the blank control. The interassay variability was found to be below 30%. HLS9 CES1 protein concentrations were normalized to the total protein amount in the sample using the unit pmol/mg protein.

### 2.6 Machine learning model development and cross-validation

A Lasso (L1-regularized) logistic regression model was developed to classify subjects across 3 tasks: (1) healthy subjects versus MASLD patients, (2) mild (NAS 1–4) versus severe (NAS 5+) MASLD, and (3) 3-class discrimination (healthy vs NAS 1–4 vs NAS 5+). Lasso logistic regression was selected over other classifiers because of the simplicity of the algorithm and the L1 penalty, which constrains model complexity by driving coefficients of uninformative features to exactly zero, thereby reducing the risk of overfitting inherent in biomarker studies with small sample sizes. Classification was performed with 3 feature sets: plasma CES1 alone, standard liver-function laboratory values alone (AST, ALT, bilirubin, WBC), and the combined feature set (plasma CES1 + labs). Hyperparameter optimization was performed via inner stratified k-fold cross-validation nested within an outer leave-one-out cross-validation (LOOCV) loop to provide unbiased performance estimates. Primary performance metrics were accuracy, F1-score (positive-class F1 for binary tasks; macroaveraged F1 for multiclass), and area under the receiver operating characteristic curve (AUC). For binary tasks, AUC was calculated for the positive class (MASLD or NAS ≥ 5). For the multiclass task, we report the macroaveraged one-vs-rest AUC based on predicted class probabilities. Feature importance was quantified as the mean absolute value of the fitted logistic regression coefficients.

### 2.7 Statistical analysis

Differences between 2 independent groups were assessed using the Mann-Whitney *U* test or Welch’s *t* test. For experiments with more than 2 groups, group differences were tested with the Kruskal-Wallis test; when the omnibus test was significant, pairwise Mann-Whitney *U* tests were performed with Bonferroni adjustment. Associations between plasma CES1 and clinical, histologic, or PK measures were summarized using two-sided Pearson or Spearman rank correlations; for adjusted analyses, partial correlations were estimated by correlating residuals from linear models of CES1 and NAS, adjusted for prespecified covariates (age, sex, BMI, diabetes status, AST, ALT). Linear trend lines in scatterplots are shown for visualization only and were not used for inference. Outliers were assessed using prespecified rules (eg, Tukey 1.5 × IQR), and sensitivity analyses excluding flagged observations are reported where applicable. Unless noted, all analyses included all participants, and data are represented as mean ± SD. A *P* value less than .05 was considered statistically significant. Computations were performed in Python 3.9.6 with NumPy, Scikit-learn, SciPy, statsmodels, and matplotlib/seaborn libraries.

## 3 Results

### 3.1 Lack of association between plasma and hepatic CES1 levels in biopsy-confirmed MASLD

**Fig. 1:**
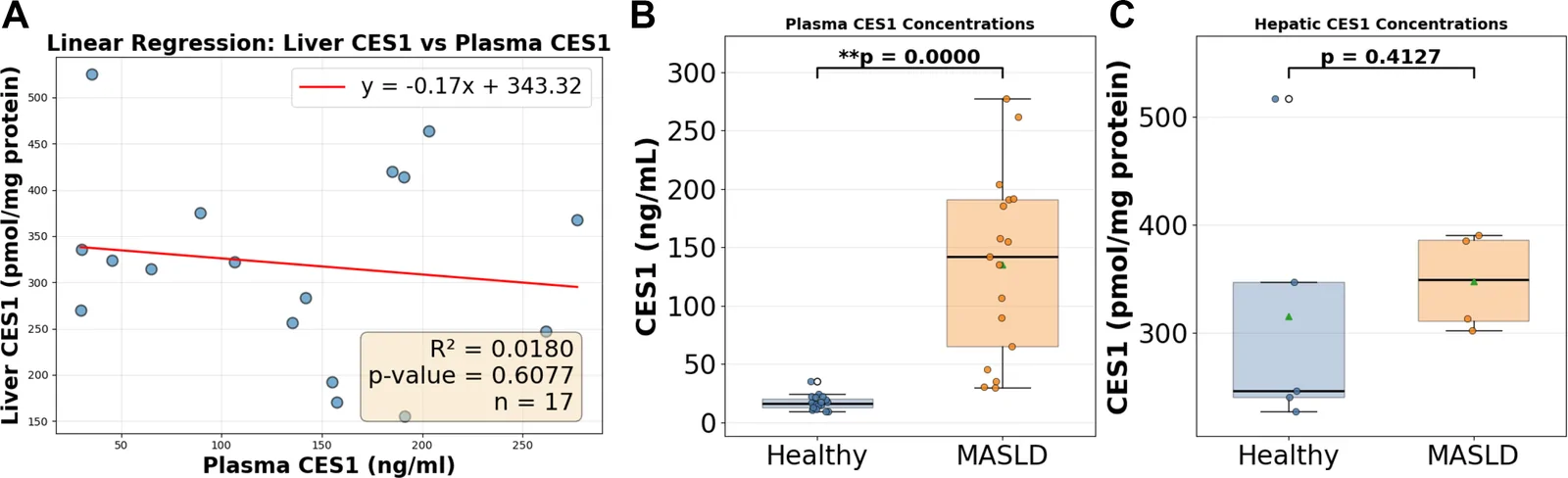
Plasma and hepatic CES1 levels in healthy and MASLD subjects. (A) Correlation between plasma and hepatic CES1 protein concentrations in MASLD patients. The scatter plot compares plasma CES1 concentrations (ng/mL) with hepatic CES1 expression measured in liver S9 fractions (pmol/mg protein) in 17 patients with biopsy-confirmed MASLD. The red line indicates the linear regression line of best fit. Comparison of CES1 concentrations in (B) plasma (ng/mL) and (C) liver S9 fractions (pmol/mg protein) between healthy subjects and patients with MASLD. Box plots display the median (horizontal line), interquartile range (box), whiskers (data range within 1.5 × IQR), mean (green triangle), and individual data points (dots). Statistical significance was assessed using the Mann-Whitney *U* test. ∗∗*P* < .0001.

In patients with biopsy-confirmed MASLD (*n* = 17), CES1 concentrations in plasma and liver S9 fractions ranged from 29.8 to 277.2 ng/mL and from 155.4 to 524.9 pmol/mg protein, respectively. Plasma CES1 showed no significant association with hepatic CES1 levels (Fig. 1A; *R*^2^ = 0.018, *P* = .608).

### 3.2 Correlation between plasma CES1 and enalapril PK and CES1 genotypes in healthy adults

**Fig. 2:**
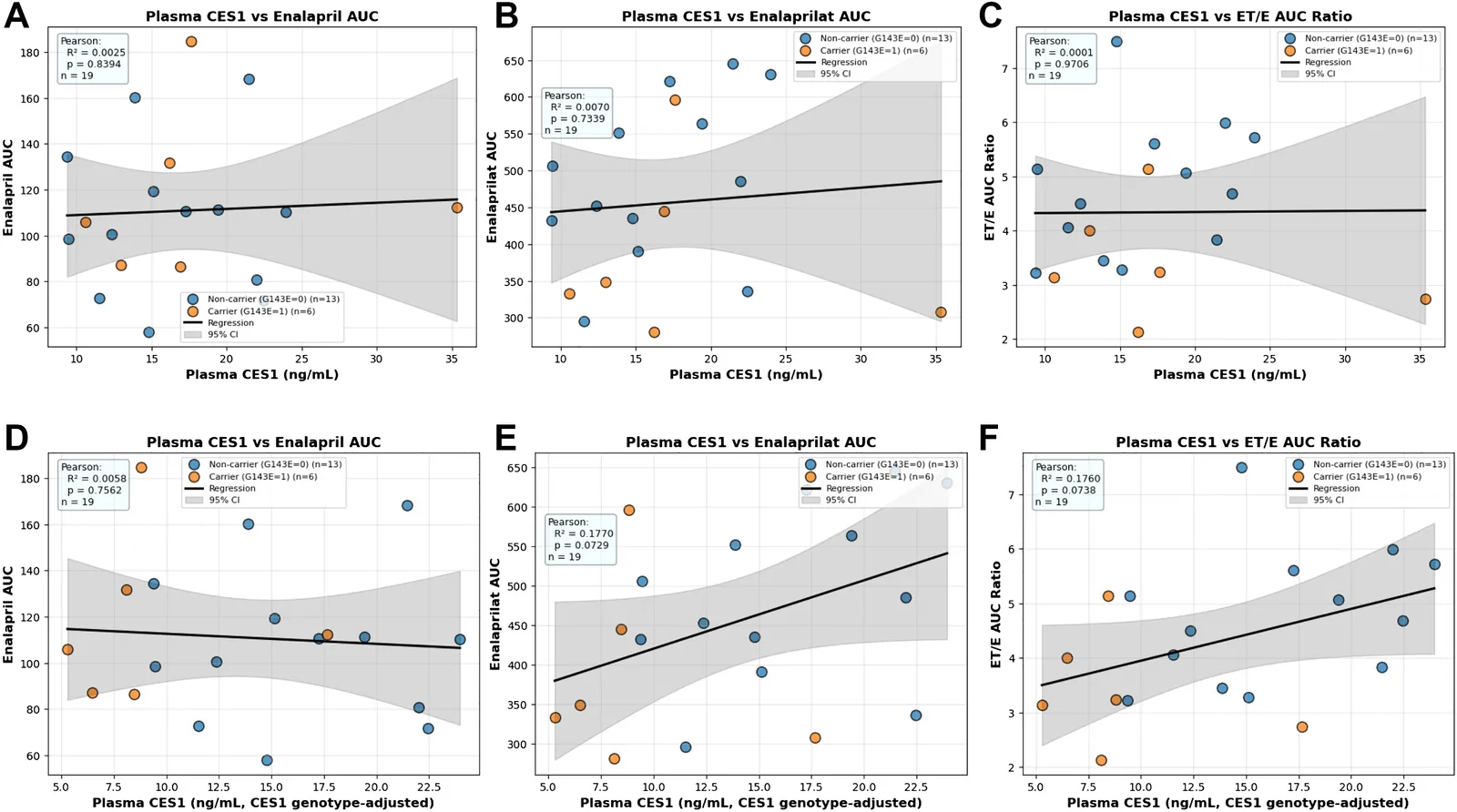
Correlations between plasma CES1 concentrations and enalapril pharmacokinetics in healthy individuals. Measured plasma CES1 versus: (A) enalapril area under the curve (AUC E), (B) enalaprilat area under the curve (AUC ET), and enalaprilat-to-enalapril AUC ratio (ET/E AUC) (C). (D–F) G143E genotype–adjusted plasma CES1 versus the same pharmacokinetic parameters as panels (A–C), where plasma CES1 concentrations were reduced by 50% in G143E heterozygous carriers to reflect reduced hepatic enzymatic function. Dashed lines represent the linear regression fit with 95% confidence bands (gray shading). Sample size (*n*) is indicated in each panel.

We analyzed data from an enalapril PK study of healthy volunteers (*n* = 19) previously conducted to determine the effect of the CES1 genetic variant G143E (rs71647871) on the PK and PD of enalapril.^4^ Plasma CES1 measured prior to enalapril administration showed no relationship with enalapril PK (Fig. 2, A–C). Linear regression analysis yielded nonsignificant results for the enalaprilat-to-enalapril AUC ratios (*R*^2^ = 0.0001, *P* = .971) (Fig. 2A), enalaprilat AUCs (*R*^2^ = 0.0070, *P* = .734) (Fig. 2B), and enalapril AUCs (*R*^2^ = 0.0025, *P* = .839) (Fig. 2C). Six of the 19 study participants were heterozygotes for the CES1 G143E genetic polymorphism. Plasma CES1 concentrations did not differ by CES1 G143E genotypes (Supplemental Fig. 1; *P* = .898).

Because G143E is a loss-of-function variant for metabolizing CES1 substrates, half of the hepatic CES1 protein in individuals who are heterozygous for G143E is nonfunctional. Accordingly, we reanalyzed the association between plasma CES1 and enalapril PK by reducing the measured CES1 concentrations in the G143E heterozygotes by 50%. Following this adjustment, Pearson correlations between plasma CES1 and enalapril PK parameters remained statistically nonsignificant (Fig. 2, D–F). However, adjusted CES1 concentrations showed a trend toward positive correlations with both the enalaprilat/enalapril AUC ratio (*R*^2^ = 0.176, *P* = .074; Fig. 2D) and enalaprilat AUC (*R*^2^ = 0.177, *P* = .073; Fig. 2E), with *P* values approaching the conventional significance threshold (ie, *P* < .05).

### 3.3 Plasma CES1 as a protein biomarker for MASLD diagnosis and severity stratification

In plasma, CES1 concentrations were markedly higher in MASLD patients than in healthy controls (Fig. 1B). Medians were 141.9 vs 16.2 ng/mL (ranges: 29.8–277.2 vs 9.4–35.3 ng/mL), an ∼8-fold difference (Mann-Whitney test: *P* < .0001, Welch’s t-test: *P* < .0001). Distributions also differed notably: healthy values clustered tightly (17.0 ± 6.3 ng/mL) with a short right tail, whereas MASLD values were broad and right-skewed (135.4 ± 78.5 ng/mL), with several individuals exhibiting concentrations greater than 200 ng/mL. By contrast, hepatic CES1 levels were comparable between the 2 groups (Fig. 1C): median 322.3 pmol/mg protein (MASLD) vs 246.5 pmol/mg protein (healthy), with overlapping ranges (155.4–524.9 vs 227.8–516.9 pmol/mg) and similar dispersion (SD: 101.9 vs 122.0). The difference in hepatic CES1 between healthy subjects and patients with MASLD was nonsignificant (Mann-Whitney: *P* = .4127; Welch’s *t* test: *P* = .6092) and remained so even after removing the outlier in the healthy group (516.9 pmol/mg) (Mann-Whitney: *P* = .1143; Welch’s *t* test: *P* = .0626).

**Fig. 3:**
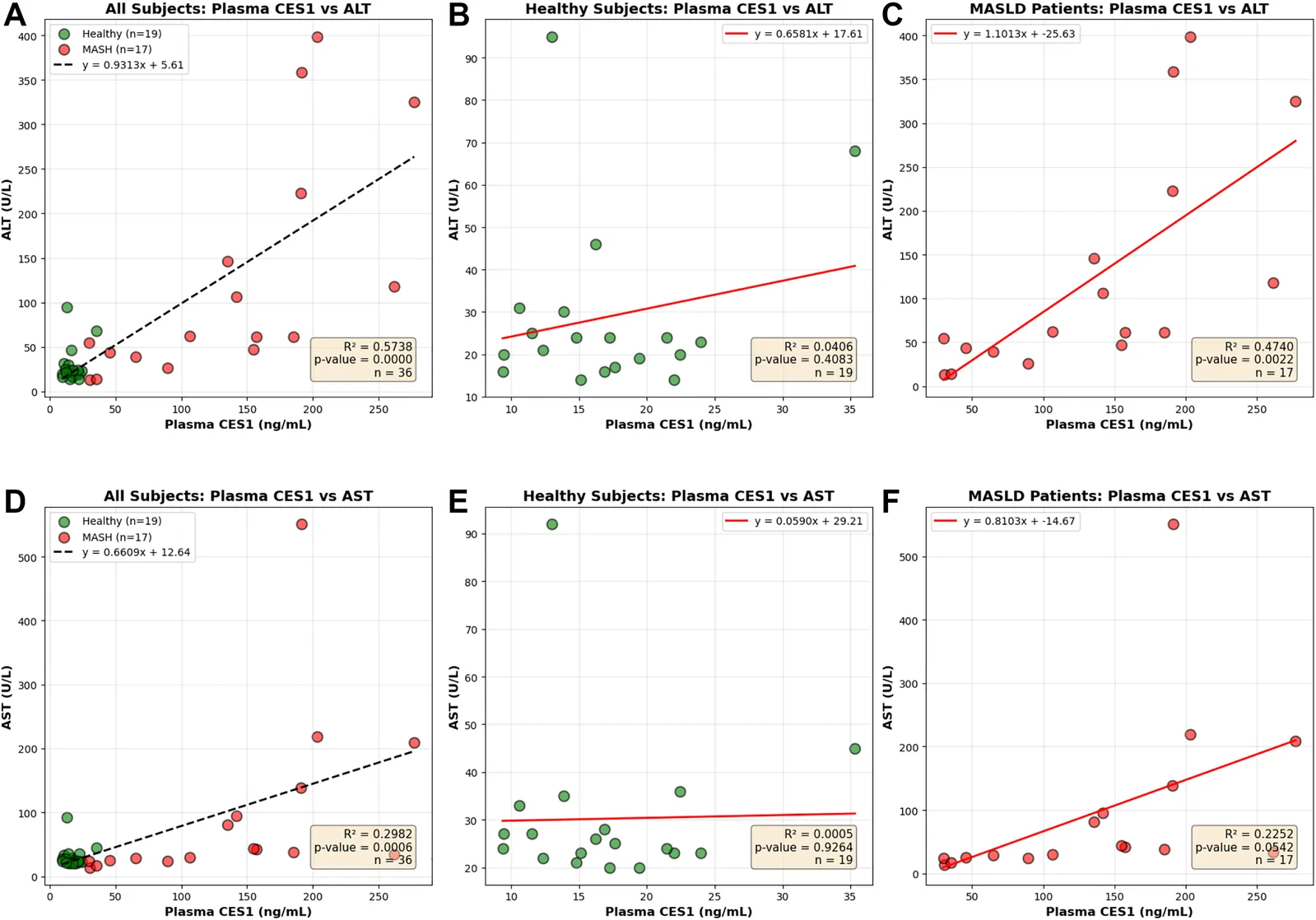
Association between plasma CES1 and liver transaminase levels stratified by disease status. (A) Plasma CES1 vs ALT in healthy subjects. (B) Plasma CES1 vs AST in healthy subjects. (C) Plasma CES1 vs ALT in MASLD patients. (D) Plasma CES1 vs AST in MASLD patients. (E) Plasma CES1 vs ALT in all subjects. (F) Plasma CES1 vs AST in all subjects. Each point represents an individual subject (green = healthy controls, red = MASLD patients). Lines indicate linear regression fits. Text boxes display the coefficient of determination (*R*^2^), *P* value, and sample size (*n*) for each analysis.

In healthy subjects (*n* = 19), no correlations were observed for either ALT (Fig. 3A; *R*^2^ = 0.041, *P* = .408) or AST (Fig. 3B; *R*^2^ = 0.001, *P* = .926). In biopsy-confirmed MASLD patients (*n* = 17), plasma CES1 was positively associated with ALT levels (Fig. 3C; *R*^2^ = 0.525, *P* = .002) and showed a positive, borderline association with AST levels (Fig. 3D; *R*^2^ = 0.252, *P* = .054). However, the positive associations should be interpreted with caution, considering the modest sample size and the influence of several high-value observations. When the 2 datasets were combined, strong correlations were observed between plasma CES1 and both ALT and AST (Fig. 3, E and F), mainly driven by significantly higher plasma CES1, ALT, and AST levels in patients with MASLD.

**Fig. 4:**
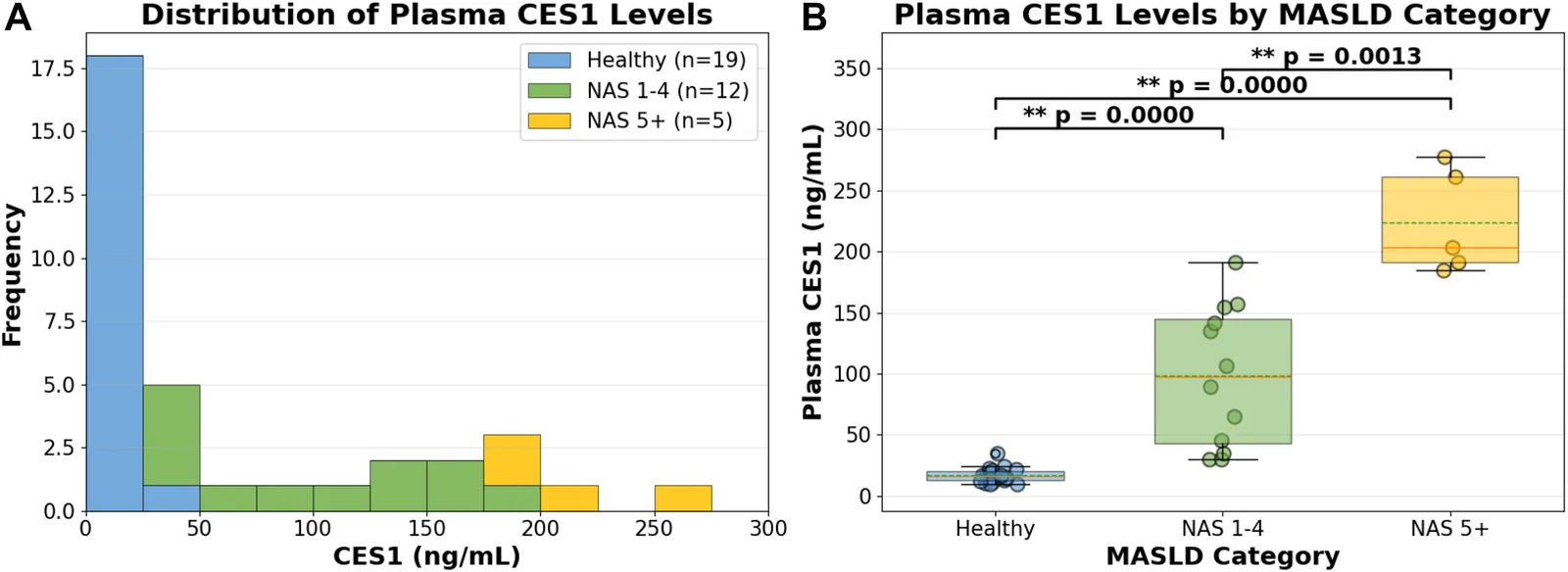
Distribution of plasma CES1 levels by MASLD activity score. (A) Stacked histogram showing the distribution of plasma CES1 concentrations across NAS categories. (B) Box plot of plasma CES1 levels stratified by NAS categories, showing median, interquartile range, mean (dashed line), and individual data points. Significance brackets indicate pairwise Mann-Whitney *U* tests with Bonferroni correction for multiple comparisons (3 comparisons, corrected *α* = 0.0167). ∗∗*P* < .01. An overall comparison was performed using the Kruskal-Wallis test.

Participants were stratified by NAS—a composite histologic index of steatosis, lobular inflammation, and hepatocellular ballooning (range 0–8; higher scores indicate greater disease severity)—into healthy controls (*n* = 19), lower activity (NAS 1–4, *n* = 12), and higher activity (NAS ≥ 5, *n* = 5) (Fig. 4). A score of 5 or higher is often used as the threshold for MASH, a severe form of MASLD. The distribution of CES1 plasma concentrations shifted rightward from healthy to NAS 1 to 4 to NAS ≥5, with tight, low values in healthy controls and broader, right-skewed values in disease groups (Fig. 4A). Medians (IQR) were 16.2 (12.7–20.4) ng/mL for healthy (*n* = 19), 97.9 (42.8–145.2) ng/mL for NAS 1 to 4 (*n* = 12), and 203.5 (190.9–261.6) ng/mL for NAS ≥5 (*n* = 5) (Fig. 4B). Overall differences were significant (Kruskal-Wallis H = 27.35, *P* < .0001). Pairwise Mann-Whitney tests with Bonferroni correction confirmed higher CES1 plasma levels in NAS 1 to 4 vs healthy (*P* < .0001), NAS ≥5 vs healthy (*P* = .0001), and NAS ≥5 vs NAS 1 to 4 (*P* = .0039).

### 3.4 Machine learning model performance comparison: CES1 versus clinical laboratory features

**Fig. 5:**
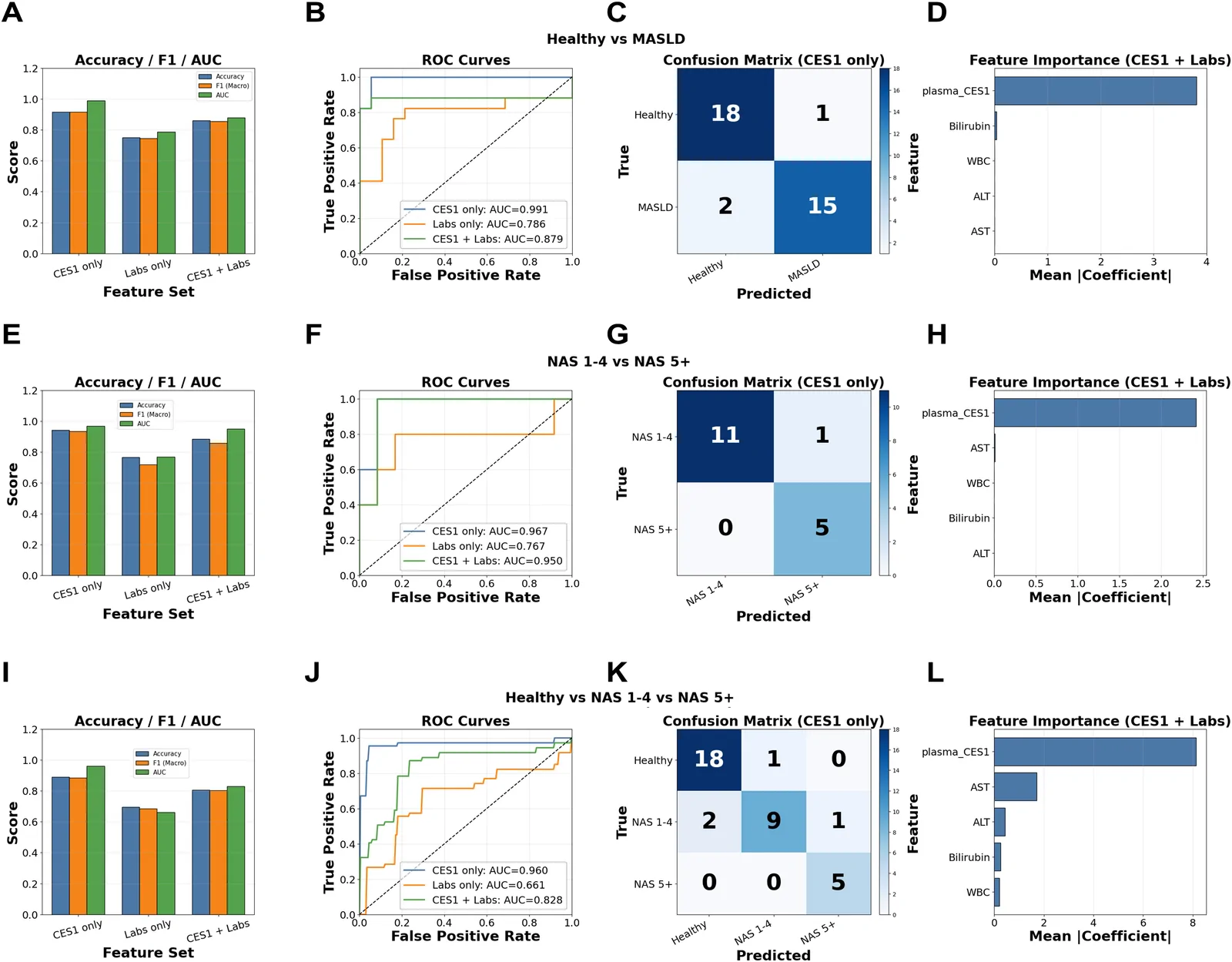
Lasso logistic regression classification of MASLD and disease severity using plasma CES1 and clinical lab features. A Lasso logistic regression model used 3 feature sets: CES1 Only (plasma CES1), Labs Only (AST, ALT, bilirubin, WBC, BMI), and CES1 and Labs combined; model performance was evaluated by LOOCV. LOOCV accuracy, F1, and AUC for each feature set (A, E, I); LOOCV ROC curves with AUC values in the legends (B, F, J); display LOOCV confusion matrices for the CES1 only feature set (C, G, K); feature importance analysis (D, H, L). Row 1 (A–D) presents the binary task (healthy vs MASLD), row 2 (E–H) presents staging within biopsy-positive disease (NAS 1–4 vs NAS ≥ 5), and row 3 (I–L) presents the multiclass task (healthy, NAS 1–4, NAS ≥ 5).

To evaluate the utility of plasma CES1 as a discriminatory biomarker, we applied a Lasso logistic regression model with nested LOOCV. Overall, plasma CES1 outperformed clinical lab features, and combining CES1 with clinical lab values did not significantly improve prediction (Fig. 5, A, B, E, F, I, and J). Using plasma CES1 as the sole feature, the model distinguished healthy subjects from MASLD patients with an AUC of 0.991 (accuracy = 92%; 18/19 healthy and 15/17 MASLD subjects correctly classified). When applied to MASLD subjects alone, plasma CES1 discriminated mild (NAS 1–4) from severe (NAS 5+) disease with an AUC of 0.967 (accuracy = 94%; 11/12 NAS 1–4 and 5/5 NAS 5+ subjects correctly classified). For the 3-class discrimination task (healthy vs NAS 1–4 vs NAS 5+), the model achieved an AUC of 0.960 (accuracy = 89%, 18/19 healthy, 9/12 NAS 1–4, and 5/5 NAS 5+ subjects correctly classified). Furthermore, plasma CES1 was identified as the dominant predictor in the feature importance analysis (Fig. 5, D, H, and L).

## 4 Discussion

### 4.1 Plasma CES1 and CES1-mediated drug metabolism

In this study, we evaluated whether plasma CES1 concentrations reflect hepatic CES1 protein levels using matched liver biopsy and plasma samples from patients with MASLD. No significant correlation was observed between hepatic and plasma CES1 levels (Fig. 1A). This dissociation is consistent with injury-induced hepatic protein release, supported by the observation that plasma CES1 concentrations in patients with MASLD were 8-fold higher than those in healthy subjects (Fig. 1B). A previous study showed that drug- and alcohol-induced liver injury increased hepatocyte-derived extracellular vesicles and enriched their cargo for liver-specific proteins—including CES1—by more than 2-fold; extracellular vesicle protein signals tracked with injury (dose/time and ALT/AST) and returned to control levels with *N*-acetylcysteine/glutathione treatment.^18^ Thus, the lack of correlation between hepatic and plasma CES1 in our biopsy cohort is likely due to plasma CES1 proteins in patients with MASLD predominantly originating from hepatic protein release driven by liver injury. However, it remains unclear whether a plasma-hepatic CES1 relationship would be detectable in healthy subjects, in whom hepatocellular integrity is preserved. These findings highlight the importance of disease context in evaluating plasma CES1 as a biomarker.

In our healthy cohort, plasma CES1 showed no association with enalapril PK (Fig. 2, A–C) when CES1 levels were not adjusted for its genotypes. Our cohort included 6 subjects who were heterozygous carriers of the CES1 loss-of-function variant G143E. When measured plasma CES1 concentrations were adjusted in G143E heterozygotes by a 50% reduction, we observed a trend toward a positive correlation with enalaprilat AUC (*R*^2^ = 0.1770, *P* = .073) and the enalaprilat-to-enalapril AUC ratio (*R*^2^ = 0.1760, *P* = .074) (Fig. 2, D–F). After factoring in the functional impact of G143E, the associations appeared more biologically coherent: higher plasma CES1 levels tracked with greater enalaprilat exposure and a higher enalaprilat-to-enalapril AUC ratio. However, these correlations were only moderate and did not reach conventional statistical significance in this small cohort. Consistent with this pattern, our prior steady-state PK study in the same cohort demonstrated that G143E carriers had a 27.4% lower enalaprilat AUC and a 32.7% lower enalaprilat-to-enalapril AUC ratio, whereas parent enalapril exposure did not differ significantly between genotypes. These findings parallel the direction observed with functionally adjusted plasma CES1, supporting the view that CES1 activity contributes to enalaprilat formation but accounts for only a limited fraction of the overall enalapril PK variability.

The discrepancy between our findings with enalapril and prior work with methylphenidate raises the possibility that the utility of plasma CES1 as a biomarker may be substrate-dependent. In a previous proof-of-concept study, we demonstrated a strong inverse correlation between plasma CES1 concentrations and *d*-methylphenidate AUC,^6^ suggesting that plasma CES1 might serve as a biomarker for hepatic CES1 expression and CES1-mediated drug metabolism. Existing evidence suggests that the impact of CES1 function on drug metabolism may be substrate-dependent. For example, pharmacogenomic studies have shown that carriers of the *CES1* G143E loss-of-function variant exhibit significant alterations in the PK of several substrates, such as clopidogrel^19^, ^20^, ^21^, ^22^ and methylphenidate,^6^^,^^23^ whereas other substrates, such as enalapril^4^^,^^24^ and trandolapril,^25^ generally did not demonstrate clinically meaningful PK differences by G143E genotypes. One explanation is that plasma CES1 predicts PK only when hydrolysis is the rate-limiting step in systemic exposure. For substrates like methylphenidate and clopidogrel, CES1 hydrolysis is critical for metabolism, making CES1 variability highly consequential. In contrast, for ACE inhibitor prodrugs, hydrolysis occurs rapidly and efficiently following absorption, such that drug exposure is determined primarily by absorption and distribution processes rather than CES1-mediated activation.^26^, ^27^, ^28^ Moreover, substrate affinity, the presence of alternative metabolic routes, and the extent of first-pass metabolism are additional factors likely to determine whether CES1 activity is rate-limiting. Taken together, these findings suggest that although plasma CES1 holds promise as a noninvasive PK biomarker, its predictive utility in drug metabolism is context-dependent and may vary substantially across different CES1 substrates.

### 4.2 Plasma CES1 as a noninvasive biomarker of MASLD

We observed an 8-fold elevation in plasma CES1 levels in patients with biopsy-confirmed MASLD compared with healthy subjects (Fig. 1B), whereas hepatic CES1 expression did not differ significantly between the MASLD and normal groups (Fig. 1C). Plasma CES1 increased stepwise with histologic activity—lowest in healthy adults, intermediate in NAS 1 to 4, and highest in NAS ≥5 (Fig. 4B). CES1 is a central regulator of hepatic lipid homeostasis. Xu et al^14^^,^^29^ showed that hepatic CES1 functions as both a triglyceride and a cholesteryl-ester hydrolase, mobilizes fatty acids for oxidation, reduces hepatic triglyceride accumulation, and modulates cholesterol metabolism through FXR signaling. Additional studies indicate that CES1 and its murine ortholog Ces1d regulate lipid droplet turnover, very low-density lipoprotein secretion, and systemic energy balance; CES1 deficiency leads to steatosis, obesity, and hyperlipidemia.^14^^,^^15^^,^^30^^,^^31^ These mechanistic data support our findings that elevations in plasma CES1 likely reflect disturbances in lipid handling and metabolic stress that characterize MASLD/MASH progression.

In machine learning analyses, we applied a Lasso (L1-regularized) logistic regression model with nested LOOCV across 3 classification tasks—binary (healthy vs MASLD), disease staging within biopsy-confirmed disease (NAS 1–4 vs NAS ≥ 5), and multiclass (healthy, NAS 1–4, NAS ≥ 5). Lasso regularization was selected because the L1 penalty constrains model complexity by driving coefficients of noninformative features to zero, making it well suited for small biomarker cohorts where overfitting is a major concern. Using plasma CES1 as the sole feature, the model achieved AUC values of 0.991, 0.967, and 0.960 for the binary, staging, and multiclass tasks, respectively (Fig. 5). Across all 3 tasks, adding routine laboratory values (AST, ALT, bilirubin, WBC) to plasma CES1 did not substantially improve classification performance, and plasma CES1 alone consistently outperformed models built on laboratory features. Our data suggest plasma CES1 captures injury/activity linked to metabolic stress, providing a distinct biological signal that could complement existing noninvasive tests in multimarker strategies for staging and monitoring. This interpretation is supported by previously published results: the population-based Rotterdam Study identified CES1 among the proteins most strongly associated with fatty liver disease in adults, and a pediatric cohort likewise reported higher serum CES1 in MASLD with positive correlations to ALT/AST and fair diagnostic performance in a binary logistic-regression model.^16^^,^^17^ Our study extends these observations by using absolute CES1 quantification and biopsy-confirmed phenotyping, providing a more direct, clinically interpretable link between plasma CES1 concentration and histologic MASLD activity.

This study has several limitations. The biopsy cohort was relatively small, and the enrolled subjects represented a population selected for diagnostic liver biopsy rather than a general MASLD population, which may limit the generalizability of the proposed CES1 thresholds. The cross-sectional design precludes evaluating temporal changes in CES1 during disease progression. In addition, the 2 cohorts differed (Table 1): paired liver-plasma samples were available only from patients undergoing biopsy for suspected MASH, whereas the PK plasma samples were obtained from healthy volunteers, introducing potential cohort-specific confounding. The healthy cohort was substantially younger (19–31 years; mean 24.6 years) than the biopsy cohort (21–70 years; mean 49.7 years), although hepatic CES1 expression does not differ between age groups in adults.^32^^,^^33^ It is noted that 6 normal liver tissues were randomly selected from a set of 153 liver samples. One donor was 1 year old, while the ages of the others ranged from 53 to 83 years. Although hepatic CES1 levels are generally lower in children, especially infants, than in adults,^32^^,^^33^ the CES1 protein level in the 1-year-old was 246.5 pmol/mg protein, similar to those in the other samples, which ranged from 227.8 to 516.9 pmol/mg protein. The difference in hepatic CES1 expression between MASLD and normal subjects remained statistically insignificant after excluding the 1-year-old subject. We found no correlation between plasma CES1 and age in either healthy or MASLD patients (Supplemental Fig. 2). Moreover, our machine learning classifier was trained and evaluated within a single cohort using internal cross-validation; independent, external validation cohorts will be required to confirm model performance and generalizability.

**Table 1:** Baseline demographic and laboratory characteristics by cohort: Healthy volunteers from an enalapril PK study and biopsy-confirmed MASLD patients from the University of Michigan Liver Tissue and Blood Repository

	Healthy Volunteers (Mean ± SD)	Patients with MASLD (Mean ± SD)
N (participants)	19	17
Age (y)	24.9 ± 3.2	47.0 ± 14.3
Height (m)	1.77 ± 0.07	1.70 ± 0.16
Weight (kg)	75.8 ± 10.7	96.49 ± 29.81
BMI (kg/m^2^)	24.0 ± 3.0	33.58 ± 10.00
AST (U/L)	30.2 ± 16.3	88.6 ± 58.1
ALT (U/L)	28.8 ± 20.5	145.9 ± 114.4
WBC	5.7 ± 1.3	6.0 ± 2.0
Total bilirubin (mg/dL)	1.02 ± 0.55	0.7 ± 0.3
Sex (M/F)	17/2	8/9

BMI, body mass index.

Beyond cohort considerations, this study has methodological limitations relevant to CES1 biomarker development. We did not systematically compare MASLD with other chronic liver diseases; therefore, additional work is needed to determine the specificity of plasma CES1 as a MASLD biomarker rather than a general marker of hepatic injury. In addition, in our prior methylphenidate study, plasma CES1 concentrations were normalized to a panel of liver-enriched proteins to account for interindividual differences in hepatic protein release rates.^6^ We did not apply such normalization in the present study because the associated improvement was marginal. Furthermore, this method requires LC–MS/MS quantification of dozens of hepatic proteins, making it infeasible in clinical settings. For these reasons, we intentionally evaluated plasma CES1 as a standalone and clinically implementable assay.

In conclusion, our findings suggest that the utility of plasma CES1 as a PK biomarker for CES1-substrate drugs may be substrate-dependent. The lack of association between plasma CES1 and hepatic CES1 levels likely reflects that plasma CES1 in patients with MASLD mainly results from injury-driven CES1 release. Importantly, our data suggest that plasma CES1 could serve as a noninvasive marker of MASLD/MASH, with the potential to complement existing fibrosis-focused indices and provide a more comprehensive assessment of disease progression.

## Conflict of interest

The authors declare no conflicts of interest.
