# Supplementary material for: Evaluating plasma carboxylesterase 1 as a protein biomarker for pharmacokinetics and metabolic dysfunction–associated steatotic liver disease
Source: Drug Metab Dispos. 2026 Apr 29;54(6):100307. doi: 10.1016/j.dmd.2026.100307 (PMC13494170; doi:10.1016/j.dmd.2026.100307)
Supplement: Supplementary file 1 [file mmc1.docx]

**Supplemental Materials**

**Evaluating Plasma Carboxylesterase 1 as a Protein Biomarker for Pharmacokinetics and** **Metabolic Dysfunction-Associated Steatotic Liver Disease**

Sun Min Jung, Pharm.D., Ph.D.^a^, Hui Yu ^a^, Xinwen Wang, Ph.D.^b^, Lucy Her, Pharm.D., Ph.D.^c^, Hao-Jie Zhu, Ph.D.^d^

^a^ Department of Pharmaceutical Sciences, University of Michigan, Ann Arbor, Michigan, USA

^b^ Department of Pharmaceutical Sciences, Northeast Ohio Medical University, Rootstown, Ohio, USA

^c^ Eli Lilly and Company, Indianapolis, IN, USA

^d^ Department of Clinical Pharmacy, University of Michigan, Ann Arbor, Michigan, USA

Corresponding Author: Hao-Jie Zhu, Ph.D.

Department of Clinical Pharmacy, University of Michigan College of Pharmacy,

428 Church Street, Room 4565, Ann Arbor, MI 48109-1065

Tel: 734-763-8449

[hjzhu@umich.edu](mailto:hjzhu@umich.edu)

ORCID: 0000-0002-2248-4419

**Supplemental Figure 1. Plasma CES1 protein concentrations by CES1 G143E genotype.** Box plots compare plasma CES1 concentrations (ng/mL, ELISA) between wild-type (G143E = 0; n = 13) and variant carriers (G143E = 1; n = 6). Points represent individual subjects; the box shows the interquartile range with the median (solid line), whiskers denote 1.5 × IQR, and the dashed green line indicates the group means. Group differences were assessed by the Mann–Whitney U test (U = 37.0, p = 0.8983)

**Supplemental Figure 2.** Correlations between ages and plasma CES1 levels in healthy subjects (left) and patients with MASLD (right).
